# Loneliness Mindsets: A New Measurement Approach and Implications for Predicting Wellbeing

**DOI:** 10.3390/bs15091196

**Published:** 2025-09-02

**Authors:** Jeni L. Burnette, Sydney Earl, Crystal L. Hoyt, Jeffrey M. Pollack

**Affiliations:** 1 Department of Psychology, North Carolina State University, Raleigh, NC 27695, USA; searl@ncsu.edu; 2 Jepson School of Leadership Studies, University of Richmond, Richmond, VA 23173, USA; choyt@richmond.edu; 3 Poole College of Management, North Carolina State University, Raleigh, NC 27695, USA; jmpolla3@ncsu.edu

**Keywords:** loneliness, mindsets, wellbeing

## Abstract

The prevalence of loneliness is rising, with both individual and societal costs, including a substantial mental health toll. Perhaps not surprisingly, given this upsurge, research focused on loneliness is proliferating. Of particular interest are the characteristics of lonely individuals and where to intercede to reduce loneliness. Interventions often focus on enhancing social skills, providing social support, offering opportunities for social interaction, and addressing maladaptive cognitions. In the current study, we seek to add to the literature on the importance of beliefs by focusing on individual differences in the meaning assigned to the nature of loneliness. Specifically, we investigate mindsets, first developing and validating a new Mindsets of Loneliness Assessment Tool (M-LAT) across two studies (*N* = 243; *N* = 382) using primarily university students. Analyses revealed four factors, which we call Lonely Attribute Mindset (LM_Attribute), Lonely People Mindset (LM_Person), Loneliness as Enhancing Mindset (LM_Enhancing), and Loneliness as Debilitating Mindset (LM_Debilitating). Lonely People and Debilitating Mindsets tended to correlate the strongest with social and psychological wellbeing. We discuss the need for future work investigating if mindset interventions targeting both of these types of mindsets can be leveraged to improve wellbeing, especially in the face of loneliness.

## 1. Introduction

Loneliness and social isolation are increasingly prevalent concerns, particularly among young adults—a trend seen globally (e.g., L. Hawkley, 2019; Snape & Manclossi, 2018). For example, in a meta-analysis of over 100,000 emerging adults, there is a significant linear increase in loneliness over a 40-year period (Buecker et al., 2021). Loneliness is defined as the perceived discrepancy between the desired and actual quality or quantity of social relationships (L. C. Hawkley, 2022; Laursen & Hartl, 2013). It is a complex emotion, encompassing anxiety and sadness as well as despair. Much of loneliness is the perception that social needs are not being met (S. Cacioppo et al., 2015; Knowles et al., 2015; Lim et al., 2016). This feeling can be evolutionarily adaptive, motivating behaviors that can improve social relations (J. T. Cacioppo & Cacioppo, 2018; L. C. Hawkley & Cacioppo, 2010). However, loneliness is also costly to wellbeing, producing greater social anxiety, reduced life satisfaction (S. Cacioppo et al., 2015), and declines in physical and mental health (C. Park et al., 2020). A systematic review of with over 2 million individuals showed that loneliness is associated with an increased risk of mortality (Wang et al., 2023). Indeed, the World Health Organization announced loneliness as a public health concern (Couto, 2023).

Considering the pervasiveness and potential detrimental consequences of loneliness, researchers and public health leaders worldwide are actively working to uncover its causes and develop effective solutions. For example, Britain appointed a loneliness minister after a report noted more than 14% of the population feels lonely, costing employers up to 3.5 billion annually (John, 2018). Additional surveys in the United States find even higher reports of loneliness with the American Psychological Association Healthy Mindsets Poll suggesting rates as high as 33% of adults reporting feeling lonely once a week over the past year (American Psychiatric Association, 2025). The loneliness epidemic, although not driven solely by the COVID-19 pandemic, was exacerbated by the associated isolation. Other contributors include the vast changes in social structures, which resulted in more disconnected societies (Putnam, 2000). Clearly, the problem of loneliness is a multifaceted issue without one clear cause, although COVID-19 and social media are often noted as primary instigators (Luhmann et al., 2023). Understanding these causes can help contribute to solutions.

In addition to societal shifts, there are individual-level psychological risk factors. The very definition of loneliness highlights that individual perception as well as subjective experiences are important components. Thus, mitigating loneliness often includes, at least in part, an effort to address cognitions that can facilitate or hinder motivation to seek out social connections. Following this line of thinking, in the current work, we home in on beliefs, or mindsets, about the nature and meaning of loneliness. For example, what do people think about the following: Is the feeling of loneliness fleeting or longer lasting? Does it represent something inherent about the person? Is it enhancing and adaptive, or debilitating and to be avoided? In the current work, we seek to understand these beliefs by offering an original tool for assessing mindsets of loneliness—namely, we develop and validate the Mindsets of Loneliness Assessment Tool (M-LAT) across two studies. In addition to developing a scale to assess beliefs, we also seek to examine how these mindsets relate to social wellbeing including social anxiety and loneliness and psychological wellbeing, including self-esteem and optimism.

### 1.1. Mindset Theory

Mindset theory differentiates between a host of beliefs, with early work focusing on fixed and growth mindsets, or the belief that human attributes, such as intelligence, are either static and unchanging, or that with hard work and the right strategies, one can develop and change their attributes (e.g., Dweck, 1999, 2006). Early theorizing also concentrated on the importance of mindsets about the nature of people for social perception (Molden & Dweck, 2006). More recently, mindset scholars extended research by examining beliefs about the meaning of experiences such as stress, with literature differentiating between enhancing mindsets, or the belief that stress is informative and an important signal that change is needed, and debilitating mindsets, or the belief that stress is negative and hinders performance (Crum et al., 2013, 2017). In the current work, to comprehensively understand loneliness mindsets, we adopt a tripartite approach to develop a new measure of mindsets of loneliness—one that examines these three interconnected beliefs about (a) the changeability of the attribute loneliness (b) the nature of people who are lonely, and (c) the experience of loneliness.

#### 1.1.1. Attribute Mindsets

Attribute mindsets are beliefs about the changeable or fixed nature of human attributes, traits, and emotions. These mindsets, first studied in the context of intelligence and academic achievement, predict a pattern of motivation and subsequent achievement. For example, individuals with growth, relative to fixed mindsets, are more likely to set goals focused on learning, deem effort a valuable tool for reaching those goals, and persist despite obstacles (Burnette et al., 2013). Considering the link to self-regulation, including help seeking, it is not surprising the theory was extended beyond the classroom to the context of understanding mental health (e.g., Schroder et al., 2016), with much of this work focused on how mindsets of emotion impact wellbeing and reactions to social stressors (e.g., Kneeland & Simpson, 2022; Schell et al., 2023). Building on this foundation, we incorporate mindsets about the potential to change the feeling of loneliness and we examine how these beliefs relate to mindsets about people who experience loneliness and the experience of loneliness itself. We then assess how these mindsets relate to individuals’ social and psychological wellbeing.

#### 1.1.2. People Mindsets

Second, we focus on beliefs about the nature of people who experience loneliness. Can lonely people change? Here, we build on a robust literature of research examining mindsets about personality and people (Molden & Dweck, 2006). Unlike mindsets about attributes which focus on the fixed or malleable nature of traits, these mindsets seek to address broader beliefs about an individual’s capacity to change their personality or who they are fundamentally as a person. We examine if these beliefs align with, or diverge from, beliefs about the malleability of loneliness as an attribute and whether they are correlated strongly enough to warrant a unified scale or an independent factor. Preliminary work suggests important distinctions. Although beliefs about the changeability of an attribute and the changeability of people with that attribute are often positively correlated, they often differentially impact outcomes (Hoyt & Burnette, 2025). For example, in recent work in the context of addiction, researchers found that mindsets about the attribute of addiction are distinct from beliefs about the nature of people with addiction, with the former being more closely tied to efficacy and treatment value, and the latter to essentialist thinking and prejudice (Burnette et al., 2024). Such findings are in line with related work on trait and social essentialism (Ryazanov & Christenfeld, 2018), which distinguishes between beliefs about attributes and beliefs about social categories as providing information about underlying essences (e.g., Rhodes et al., 2012). Although these are often positively correlated, they differentially predict outcomes (Hoyt et al., 2024; Haslam et al., 2000; Levy et al., 1998). Given the research demonstrating that beliefs about an attribute and about people with that attribute are related yet can differentially predict outcomes, we examine how these patterns apply to loneliness beliefs and wellbeing.

#### 1.1.3. Experience Mindsets

In addition to mindsets about attributes and people, the third component of our framework focuses on experience mindsets, which are rooted in an individual’s beliefs about the meaning assigned to experiences such as stress, failure, and struggle (e.g., Becker et al., 2023; Crum et al., 2013; Tao et al., 2022). These mindsets are broken down into enhancing and debilitating, rather than growth and fixed and they influence both behavioral and psychological outcomes. For example, those with an enhancing mindset tend to see stress, failure, or struggle as a motivator that drives performance, leading to more adaptive coping strategies, increased resilience, and improved wellbeing (Crum et al., 2013, 2017; Huebschmann & Sheets, 2020). Conversely, individuals with a debilitating mindset often perceive these same experiences as harmful, which can lead to avoidance behaviors, anxiety, and decreased performance. Overall, experience mindsets play a crucial role in shaping how individuals interpret and respond to stress, failure, and struggle, with enhancing mindsets fostering resilience and debilitating mindsets undermining wellbeing.

We suggest that loneliness is also an experience that can be viewed as enhancing or debilitating. On the one hand, evolutionary theories of loneliness highlight how it is an adaptive signal, such as thirst, that can encourage seeking out greater social connections and thus, it is a learning tool necessary for ensuring group cohesion and community (J. T. Cacioppo & Cacioppo, 2018). Additionally, philosophers discuss loneliness, or solitude, as an opportunity for self-reflection and personal growth, seeing it as a state that allows individuals to engage deeply with their inner selves and cultivate a sense of individuality as well as creativity, spirituality, and freedom (Long & Averill, 2003). On the other hand, loneliness can cause cognitive and emotional impairments such as heightened anxiety, depressive symptoms, and a hyper-focus on social rejection cues, all of which may exacerbate the state of loneliness (J. T. Cacioppo & Hawkley, 2009; Eccleston & Crombez, 1999; Gow et al., 2007; L. C. Hawkley & Cacioppo, 2010). Whether loneliness is believed to be an experience that is beneficial or harmful may depend largely on how someone interprets and makes meaning of time spent alone (Laursen & Hartl, 2013). In the current work, we are interested in people’s beliefs about the meaning of experiencing loneliness. We explore if these beliefs represent opposite ends of a single continuum or coexist at varying strengths within individuals. Furthermore, we examine how these beliefs relate to mindsets of the attribute and people with loneliness and to wellbeing.

#### 1.1.4. Mindsets and Social and Psychological Wellbeing

In addition to using the tripartite approach for developing a new measure of loneliness mindsets, we also seek to investigate how the different facets of mindsets relate to social wellbeing, including social anxiety and feelings of loneliness (i.e., frequency, personal identification with loneliness), as well as indicators of psychological wellbeing, including a general sense of wellbeing, self-esteem, and optimism. Wellbeing is a broad construct that encompasses not only the absence of negative states like distress or social anxiety but also the presence of positive psychological resources such as self-esteem and optimism (e.g., Ryff, 1989; Seligman, 2002). Both social and psychological wellbeing contribute to an individual’s ability to cope with challenges and maintain a positive outlook on life, which are essential components of overall mental health and life satisfaction. We expect growth mindsets and enhancing mindsets to be negatively linked to social anxiety and loneliness but positively linked to generalized wellbeing and related concepts such as self-esteem and optimism. For example, growth attribute mindsets about anxiety and emotions are correlated negatively with psychological distress and anxiety (e.g., Burnette et al., 2020; Schroder et al., 2019; Valentiner et al., 2011). Furthermore, growth mindsets of people correlate positively with wellbeing, including self-esteem and general optimism (e.g., Karaman et al., 2018). Growth mindsets of both attributes and people can foster general wellbeing and resilience, encouraging individuals to view challenges as opportunities rather than threats, which promotes both self-esteem and optimism.

Furthermore, believing that potentially difficult psychological experiences, such as stress or struggle, are enhancing, relative to debilitating, can be a protective factor and enhance wellbeing (L. Chen & Yang, 2022). For example, a stress-is-enhancing mindset attenuated the relationship between perceived stress and mental health concerns (Huebschmann & Sheets, 2020). Additionally, whereas an enhancing mindset is related negatively to destructive emotions but positively to self-efficacy, a debilitating mindset is positively linked to negative affect, greater attentional bias to negative feedback, and lower cognitive flexibility (Becker et al., 2023; Crum et al., 2017). Furthermore, debilitating mindsets are positively linked to feelings of stress in the face of adverse events (D. Park et al., 2018). We expect similar relations in the current work examining experience mindsets of loneliness and relations with both social and psychological wellbeing.

#### 1.1.5. Synthesis

There are two primary aims in the current work. First, we seek to develop a comprehensive measure of loneliness mindsets that captures the nuanced beliefs individuals hold about loneliness (i.e., Mindsets of Loneliness Assessment Tool; M-LAT). We seek to establish factor structure and examine association with other highly studied mindset measures and personality constructs. We propose that the M-LAT will capture distinct facets of loneliness beliefs—attribute, people, and experience. We also expect that loneliness mindsets will be moderately related to established measures of mindset beliefs in conceptually linked areas such as shyness (Valentiner et al., 2011) and emotions (Tamir et al., 2007) and broader contexts such as people (e.g., Chiu et al., 1997) and intelligence (Dweck, 1999). Though mindsets vary across domains (Dweck & Leggett, 1988) and research suggests that these beliefs are distinct, they may also share an underlying global dimension, or “core mindset” (Schroder et al., 2016). Thus, we expect positive, moderate correlations. We also explore links between mindsets and demographics, although existing work is mixed and inconclusive (Degol et al., 2018; Destin et al., 2019; Sisk et al., 2018). Second, we seek to use this newly developed assessment and its factors to examine links between mindsets and key indicators of social and psychological wellbeing including social anxiety, loneliness, general wellbeing, self-esteem, and optimism. Understanding which beliefs predict these outcomes can contribute to future theory and intervention development.

## 2. Methods: Study 1

Our primary objective in Study 1 is to develop items and establish a preliminary structure for the M-LAT. We also explore links between the factors and social wellbeing, with a focus on social anxiety and loneliness.

### 2.1. Participants

We recruited participants via an introductory psychology course at a large Southeastern University in the United States in 2024. Students received one research credit for participating. After removing duplicates, those who did not consent or who failed the comprehension or attention checks, and those with 50% or more missing data (e.g., Hoyt et al., 2024; Peer et al., 2022), we had a final sample of 243 participants, which provided adequate power based on recommendations requiring 200 to 250 participants (Sakaluk & Short, 2017). The sample was nearly evenly split between men (*n* = 120) and women (*n* = 119). Age ranged from 18 to 25 (M = 19.30; SD = 1.39). See Table S1 (in Supplemental) for more details on demographic characteristics.

### 2.2. Procedure

All study materials were delivered to participants online via Qualtrics. After consenting, participants first completed the M-LAT and then were asked to respond to the additional measurements. Finally, participants responded to demographic questions^1^. At the completion of the survey, participants were provided with resources for coping with and overcoming loneliness, including a link to a blog post on The Jed Foundation’s website (Burch, n.d.) and information about support services available at the university where the study was conducted. Throughout the survey, we included a comprehension check (i.e., “To show that you are paying attention, please leave this question blank”; Hoyt et al., 2024) and attention checks in line with best practices for helping to improve data quality (i.e., “I see myself as someone who did not read this statement”; Peer et al., 2022). All participants provided informed consent for inclusion before they participated in the study. The study was conducted in accordance with the Declaration of Helsinki, and the protocol was approved by the Ethics Committee (IRB #: 26497).

### 2.3. Measures

#### 2.3.1. Mindsets of Loneliness

Using focus groups, we initially developed 91 items to capture mindset-related beliefs relevant to loneliness. These items included changeability beliefs about loneliness as an attribute, being a lonely person, as well as the experience of loneliness as being enhancing or debilitating. From this original pool, items were retained based on theoretical relevance and uniqueness. Ultimately, 30 items were selected for further testing. Participants responded to these 30 items using a Likert scale ranging from 1 (strongly disagree) to 7 (strongly agree). Results from an EFA, as well as reliabilities, are below in the analysis section.

#### 2.3.2. Mindsets of Intelligence

The 6-item Mindsets of Intelligence scale (Dweck, 1999) measures the extent to which individuals consider their intelligence to be fixed or malleable on a scale ranging from 1 (strongly disagree) to 7 (strongly agree). Three items were recoded so that higher scores indicated a stronger growth mindset of intelligence (e.g., “You can always improve your intelligence substantially”; α = 0.92).

#### 2.3.3. Mindsets of Shyness

The 4-item Mindsets of Shyness scale (Valentiner et al., 2011) measures the extent to which individuals consider inhibited social behavior to be fixed or malleable on a scale ranging from 1 (strongly disagree) to 7 (strongly agree). All items were recoded so that higher scores indicated a stronger growth mindset of shyness (e.g., “Your level of shyness is something about you that you can’t change very much; α = 0.67).

#### 2.3.4. Social Anxiety

The 4-item Mini Social Phobia Inventory (SPIN; Connor et al., 2001) is a brief self-rated screening instrument for generalized social anxiety disorder utilizing a scale ranging from 1 (not at all) to 5 (extremely), with higher scores indicating greater social anxiety (e.g., “I avoid activities in which I am the center of attention”; α = 0.86).

#### 2.3.5. Loneliness

We assessed loneliness with two single item questions. First, we measured loneliness frequency (i.e., “How much loneliness are you experiencing in your life right now?”). Responses to each item were rated on a scale ranging from 1 (none) to 7 (an extreme amount). Second, identification with being lonely was measured with a single item (i.e., “Are you a lonely person?”) with responses of Yes/No and coded such that 0 = No and 1 = Yes.^2^

### 2.4. Analytical Approach

All analyses were conducted in SPSS 29. First, we conducted an Exploratory Factor Analysis (EFA) using maximum likelihood (ML) estimation and promax rotation. ML is one of the most robust extraction methods with the advantage of providing generalizable results when conducting subsequent confirmatory factor analyses (CFA; Fabrigar et al., 1999; Nunnally & Bernstein, 1994). Additionally, promax rotation allows factors to correlate and has been shown to be one of the more robust rotations (Carpenter, 2018). We utilized an iterative approach, conducting several EFAs to establish the factor structure of the M-LAT (Beavers et al., 2013; Henson & Roberts, 2006). We excluded items based on low communalities (≤0.40) and factor loadings (≤0.40; cross-loadings or absence of loading; Carpenter, 2018) at each step with a focus on recommendations to pursue simple structure that can be most easily interpreted and replicated (e.g., Sakaluk & Short, 2017). We repeated this until no additional items needed to be removed. Factors were determined by the scree plot (number of extracted factors before the second elbow of the plot; e.g., Beavers et al., 2013) and Kaiser criterion (number of extracted factors with eigenvalues above 1; Gorsuch, 2014). For the final iteration, we calculated Cronbach’s internal reliability for each factor. Once the M-LAT factor structure was established and internal reliability was assessed, we calculated descriptive statistics as well as correlations with the mindset scales for convergent validity and then examined links to outcomes for predictive validity.

## 3. Results: Study 1

### 3.1. Exploratory Factor Analysis

The initial solution in our EFA produced six factors as demonstrated by the scree plot (McCrosky & Young, 1979) and eigenvalues greater than one (Kaiser, 1960). There were five items with communalities < 0.40. After removal of these items, we conducted a second EFA, which resulted in a five-factor solution and one item with a communality < 0.40. After removing this item, we ran a final EFA on the remaining 23 items resulting in a five-factor solution (see Figure S1 in Supplemental for scree plot). Following this analysis, each item had communalities >0.40 and there were no cross-loadings. An additional eight items were removed based on distinctiveness. Two of the factors had thematic overlap and were theoretically similar. Additionally, they were significantly and highly correlated (*r* = 0.71, *p* < 0.001). Thus, we combined these two factors into one.

These procedures resulted in four distinct factors comprising 16 items (see Table 1). These distinct factors include beliefs regarding the attribute of loneliness (four items, e.g., “I will always be lonely and there isn’t much I can do to change that”), being a lonely person (six items, e.g., “Being lonely is an inherent part of who I am”) and perceiving loneliness as enhancing (three items, e.g., “When I experience loneliness, I think it can help me grow”) or debilitating (three items, e.g., “Loneliness makes me feel unmotivated”). We label these factors Lonely Attribute Mindset (LM_Attribute), Lonely People Mindset (LM_Person), Loneliness as Enhancing Mindset (LM_Enhancing), and Loneliness as Debilitating Mindset (LM_Debilitating). Each scale had good reliability (α = 0.80 to 0.90). Additionally, absolute correlations among factors ranged from 0.00 to 0.42 (see Table 2).

### 3.2. Construct Validity

We first calculated correlations between scores on each of the four factors of mindsets of loneliness with mindsets of intelligence and mindsets of shyness (see Table 3). LM_Attribute, LM_Person, and LM_Enhancing correlated significantly and positively with growth mindsets of intelligence (*r* = 0.18 to 0.26, *p*s < 0.001). LM_Person correlated significantly and positively with growth mindsets of shyness (*r* = 0.36; *p* < 0.001). Surprisingly, LM_Debilitating also correlated significantly and positively with growth mindsets of shyness (*r* = 0.15; *p* = 0.02). We also calculated correlations between mindset factors and demographic characteristics including age, school year, gender, and self-reported socioeconomic status (see Table 3). Across all factors and demographic characteristics, only LM_Debilitating correlated significantly with gender such that men reported stronger debilitating beliefs of loneliness compared to women (*r* = 0.21, *p* < 0.001).

### 3.3. Predictive Validity

We looked at the correlations between mindset factors, social anxiety, and loneliness (see Table 3). LM_Person, LM_Enhancing, and LM_Debilitating were significantly related to social anxiety. A stronger growth mindset about lonely people was negatively related to social anxiety (*r* = −0.29, *p* < 0.001), whereas stronger beliefs that loneliness is enhancing (*r* = 0.13, *p* = 0.05) or debilitating (*r* = 0.26, *p* < 0.001) were both positively related to social anxiety. LM_Person and LM_Debilitating were significantly related to levels of loneliness, such that a stronger growth mindset of lonely people was negatively related to the frequency of loneliness (*r* = −0.29, *p* < 0.001) and a stronger debilitating belief of loneliness was positively related to the frequency of loneliness (*r* = 0.31, *p* < 0.001). Additionally, LM_Attribute, LM_Person, and LM_Debilitating were significantly correlated with identifying as being a lonely person. A stronger growth mindset regarding the attribute of loneliness (*r* = −0.17, *p* = 0.007) and being a lonely person (*r* = −0.34, *p* < 0.001) correlated negatively with identifying as being a lonely person. A stronger debilitating belief of loneliness was positively linked to identifying as a lonely person (*r* = 0.21, *p* = 0.001). In line with past work, social anxiety was also linked in expected ways to loneliness (*r* = 0.36, *p* < 0.001).

## 4. Discussion: Study 1

Study 1 established a preliminary structure for the measurement of mindsets of loneliness. We initially developed 30 items to capture the mindset-related beliefs relevant to loneliness, including beliefs related to the attribute of loneliness, being a lonely person, and the experience of loneliness itself. To assess these items, we conducted several iterative EFAs utilizing ML and promax rotation to arrive at a preliminary solution of four factors and 16 items. These distinct factors include beliefs regarding the attribute of loneliness (LM_Attribute) and being a lonely person (LM_Person) as well as perceiving loneliness as enhancing (LM_Enhancing) and debilitating (LM_Debilitating).

We also examined links to social anxiety and loneliness. A stronger growth mindset of the attribute of loneliness (LM_Attribute) was not significantly linked to social anxiety or loneliness; however, as expected, a stronger growth mindset of lonely people (LM_Person) was negatively linked to social anxiety and loneliness. Both an enhancing mindset about loneliness (LM_Enhancing) and a debilitating mindset about loneliness (LM_Debilitating) were positively associated with social anxiety; however, only a debilitating mindset was linked to greater loneliness. Although most of these findings match hypotheses, there are a couple of surprising findings. First, attribute mindsets did not relate to social anxiety and loneliness. Rather, the nature of people with the attribute, in this case lonely people, revealed stronger relations to these outcomes. Additionally, we did not expect enhancing mindsets to be positively linked to social anxiety. One possibility is that individuals who try to reframe loneliness as meaningful may still experience social distress, potentially due to increased self-focus. This aligns with work showing that cognitive reappraisal strategies can sometimes coexist with elevated emotional sensitivity (Tugade & Fredrickson, 2004). Before drawing conclusions about unexpected findings, we wait for results in Study 2, where we seek to replicate the main findings.

In Study 2, we extend Study 1 in four main ways. First, we conducted a CFA to confirm the structure of the measure. Second, we examined links between our newly developed measure and two different mindsets—namely, mindsets of people and mindsets of emotion. Third, we added the Big Five Personality (Gosling et al., 2003), as mindsets overlap with these stable individual differences and these traits are associated with loneliness (Buecker et al., 2020). Including these variables allows us to assess discriminant validity by testing whether loneliness mindsets are conceptually distinct from personality traits, and to examine whether mindsets predict outcomes above and beyond broad personality traits. Prior research looking at personality correlates tends to find negative links between growth mindsets and neuroticism and smaller less robust links among other personality constructs such as extroversion (Satchell et al., 2017; Zirenko, 2018). Furthermore, many of the Big Five traits are closely tied to loneliness, with neuroticism showing the strongest links, likely due to the tendency for people higher in neuroticism to experience negative emotions, to interpret things more negatively, and to engage in more maladaptive coping (Buecker et al., 2020). Fourth, we replaced the single item loneliness measures with a longer, validated, measure and we added general measures of psychological wellbeing, self-esteem, and optimism (Rosenberg, 1965; Scheier et al., 1994).

## 5. Study 2

Our objectives in Study 2 were to confirm the structure for the M-LAT, examine additional variables for construct validity, replicate links to loneliness using a longer more valid measure, and assess predictive validity through exploring relations with psychological wellbeing, including self-esteem and optimism.

### 5.1. Participants

We aimed for a sample size of at least 400 participants in order to have adequate power to run the CFA (Gorestzko et al., 2021). We recruited approximately half of our participants from an introductory psychology course at a large Southeastern University in the United States. We recruited the other half from CloudResearch (Hartman et al., 2023), one of the more frequently used online panel providers of participants (Douglas et al., 2023). Students received one research credit for participating, and CloudResearch participants received USD 1.00 for completing the survey. After removing duplicates, those who did not consent, failed the comprehension or attention checks, and with 50% or more missing data (e.g., Hoyt et al., 2024; Peer et al., 2022), we incorporated 154 participants from the university sample and 228 from CloudResearch, resulting in a final sample of 382 participants. The sample was evenly split between men (*n* = 195) and women (*n* = 174). Age ranged from 18 to 24 (M = 21.06; SD = 2.30). See Table S1 for more details on demographic characteristics.

### 5.2. Procedure

All study materials were delivered to participants online via Qualtrics. After consenting, participants first completed the M-LAT and then were asked to respond to the additional measurements. At the end of the survey, participants responded to demographic questions. In terms of the debrief, at the conclusion of the survey participants were provided with the same Jed Foundation resources from Study 1 related to coping with, and overcoming, loneliness. As in Study 1, throughout the online survey, we included comprehension checks (i.e., “To show that you are paying attention, please leave this question blank”; Hoyt et al., 2024) and attention checks in line with best practices for screening of quality data (i.e., “I see myself as someone who did not read this statement”; Peer et al., 2022). All participants provided informed consent for inclusion before they participated in the study. The study was conducted in accordance with the Declaration of Helsinki, and the protocol was approved by the Ethics Committee (IRB #: 26497).

### 5.3. Measures

#### 5.3.1. Mindsets of Loneliness

We used the final 16 items from the iterative EFA in Study 1. Participants completed these on a scale ranging from 1 (strongly disagree) to 7 (strongly agree). The LM_Attribute and LM_Person factors were coded such that a higher score indicates a stronger growth mindset. The LM_Enhancing and LM_Debilitating factors were coded such that a higher score indicates stronger enhancing and debilitating beliefs, respectively. Reliability for the factors ranged from 0.78 to 0.92 and correlations ranged from 0.07 to 0.52 (see Table 4).

#### 5.3.2. Mindsets of People

The 8-item Mindsets of People scale (Chiu et al., 1997; Dweck, 1999) measures the extent to which one believes that people can change or that they cannot change their basic characteristics on a scale ranging from 1 (strongly disagree) to 7 (strongly agree). Four items are growth worded and four items are fixed. We recoded such that higher scores indicate stronger growth mindsets (e.g., “Everyone, no matter who they are, can significantly change their basic characteristics”; α = 0.87).

#### 5.3.3. Mindsets of Emotion

The four-item Mindsets of Emotion scale (Tamir et al., 2007) measures the extent to which individuals consider emotion to be a fixed or malleable attribute on a scale ranging from 1 (strongly disagree) to 7 (strongly agree). Two items are growth-worded and two are fixed. We recoded such that higher scores indicate a stronger growth mindset of emotion (e.g., “If I want to, I can change the emotions that I have”; α = 0.83).

#### 5.3.4. Big Five Personality

The Ten-Item Personality Inventory (TIPI) assesses the “Big Five” personality traits, including extraversion, agreeableness, conscientiousness, emotional stability, and openness to experience (Gosling et al., 2003) on a scale ranging from 1 (strongly disagree) to 7 (strongly agree). Higher scores indicate a stronger tendency to exhibit a given trait. Reliability ranged from 0.43 to 0.73. Importantly, low reliability is common and expected for the TIPI as a result of only two items capturing broad concepts (Gosling et al., 2003).

#### 5.3.5. Loneliness

We assessed loneliness with the validated UCLA loneliness scale (Russell et al., 1978), which consists of 20 items rated on a scale ranging from 1 (strongly disagree) to 7 (strongly agree). Higher scores indicate more loneliness (e.g., “I feel completely alone”; α = 0.96).

#### 5.3.6. General Wellbeing

We incorporated a generalized wellbeing measure (Diener et al., 1985), which consists of five items rated on a scale ranging from 1 (strongly disagree) to 7 (strongly agree). Higher scores indicate greater wellbeing (e.g., “In most ways my life is close to my ideal”; α = 0.91).

#### 5.3.7. General Self-Esteem

We used a generalized self-esteem measure (Rosenberg, 1965), which consists of 10 items rated on a seven-point Likert scale ranging from 1 (strongly disagree) to 7 (strongly agree). Higher scores indicate more self-esteem (e.g., “I feel that I am a person of worth”; α = 0.91).

#### 5.3.8. General Optimism

To measure optimism, we used the 10-item Life Orientation Test (Scheier et al., 1994), which includes four filler items. The items are rated on a seven-point Likert scale ranging from 1 (strongly disagree) to 7 (strongly agree). Higher scores indicate more optimism (e.g., “In uncertain times, I usually expect the best”; α = 0.84).

### 5.4. Analytical Approach

All analyses were conducted in SPSS 29 or AMOS 29. First, we conducted a CFA which is essential for validating measurement instruments, as it rigorously tests whether the data align with the hypothesized factor structure, thereby confirming the theoretical constructs being measured. A CFA provides a comprehensive assessment of model fit, enabling the identification and correction of potential issues, which enhances the precision and reliability of the measurement tool (Hu & Bentler, 1999). We assessed the fit of the CFA model using several indices, including the Akaike Information Criterion (AIC; Akaike, 1974), Bayesian Information Criterion (BIC; Schwarz, 1978), Comparative Fit Index (CFI; Bentler, 1990), Tucker–Lewis Index (TLI; Tucker & Lewis, 1973), and Root Mean Square Error of Approximation (RMSEA; Steiger, 1998). A good model fit is generally indicated by a CFI and TLI greater than 0.90. RMSEA values below 0.05 indicate good fit, whereas values below 0.08 represent an acceptable fit (Murphy et al., 2020). Additionally, we used the χ^2^ statistic to evaluate model fit. Although a non-significant χ^2^ (*p* > 0.05) typically indicates good fit, this statistic is highly sensitive to large sample sizes (Byrne, 2001). Therefore, when the χ^2^ statistic was significant, we relied more heavily on the other fit indices to determine overall model acceptability. Second, we report simple correlations among constructs before running a hierarchical regression, entering covariates in Step 1 and each mindset factor in Step 2. There were no multicollinearity problems among the predictors, as indicated by tolerance values greater than 0.10 and Variance Inflation Factor (VIF) scores that were all less than 10 (Mertler & Vannatta, 2002). All other assumptions were met.

## 6. Results: Study 2

### 6.1. Confirmatory Factor Analysis

**Figure 1:**
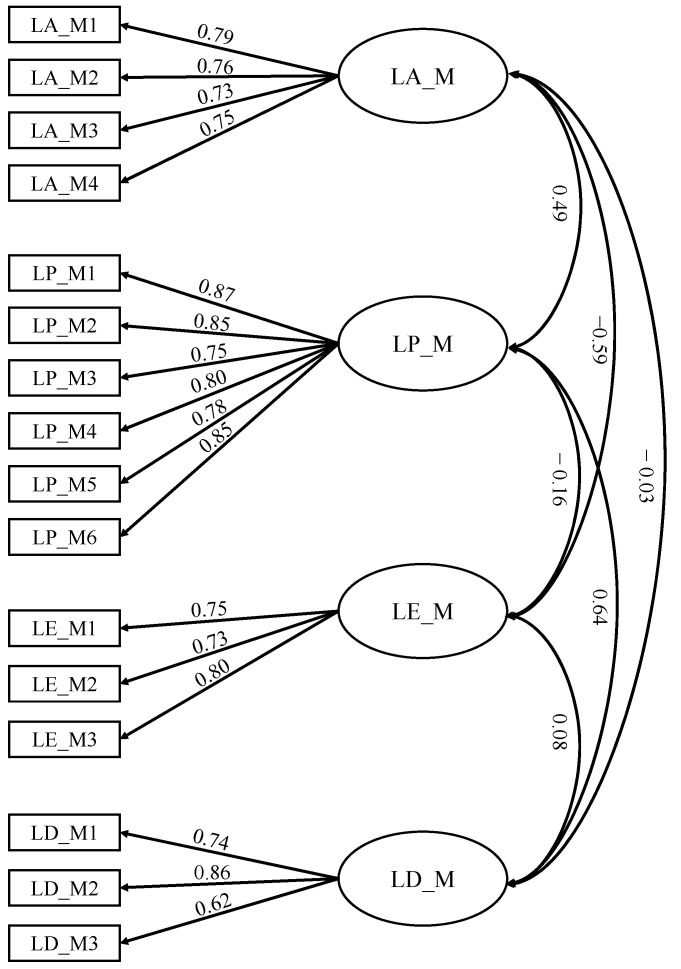
Factor structure with loadings and correlations.

The preliminary four-factor, 16-item M-LAT scale was first tested with a CFA (see Figure 1). The model showed adequate fit, RMSEA = 0.08, CFI = 0.94, TLI = 0.91, and χ^2^ = 319.56 (*p* < 0.001). Factor loadings ranged from 0.62 to 0.87 (See Table 1). Each scale had good reliability (α = 0.78 to 0.92) and absolute correlations ranged from 0.07 to 0.52 (see Table 2). 

### 6.2. Construct Validity

We first calculated correlations between scores on each of the four factors of mindsets of loneliness with mindsets of people and emotion (see Table 4). LM_Attribute, LM_Person, and LM_Enhancing correlated significantly and positively with mindsets of people (*r*s = 0.10 to 0.22, *p*s < 0.05), whereas LM_Debilitating correlated significantly and negatively with mindsets of people (*r* = −0.17, *p* < 0.001; see Table 4). Similarly, LM_Attribute, LM_Person, and LM_Enhancing correlated significantly and positively with mindsets of emotion (*r* = 0.14 to 0.45, *p*s < 0.001), whereas LM_Debilitating correlated significantly and negatively with mindsets of emotion (*r* = −0.44, *p* < 0.001; see Table 4). We also calculated correlations between mindset factors and the Big Five personality traits as well as demographic characteristics including age, gender, and self-reported socioeconomic status (see Table 4). LM_Attribute, LM_Person, and LM_Debilitating significantly correlated with each of the Big Five personality traits, ranging from 0.14 to 0.48, with emotional stability being most highly correlated. LM_Enhancing did not significantly correlate with any of the personality traits. LM_Attribute and LM_Person were correlated significantly and negatively with age, such that younger participants reported a stronger growth mindset (*r*s = −0.30; −0.18; *p*s < 0.001, respectively). LM_Debilitating was correlated significantly and negatively with gender (*r* = −0.13; *p* = 0.010), such that men were more likely to report a stronger debilitating mindset of loneliness. Finally, LM_Attribute and LM_Person correlated significantly and negatively with self-reported socioeconomic status (*r*s = −0.23; −0.19; *p* < 0.001, respectively), while LM_Debilitating correlated significantly and positively (*r* = 0.11; *p* = 0.037), such that a lower self-reported socioeconomic status was related to stronger growth mindsets and a weaker debilitating mindset of loneliness.

### 6.3. Predictive Validity

We first report simple correlations. Loneliness is significantly and negatively correlated with LM_Attribute and LM_Person mindsets (*r*s = −0.26 and −0.67, respectively, *p*s < 0.001); whereas a LM_Debilitating mindset is significantly and positively correlated (*r* = 0.60; *p* < 0.001). Additionally, wellbeing is significantly and positively correlated with LM_Attribute and LM_Person mindsets (*r*s = 0.29 and 0.39, respectively; *p*s < 0.001), whereas LM_Debilitating mindset is significantly and negatively correlated (*r* = −0.36; *p* < 0.001). Similarly, optimism and self-esteem are correlated positively with LM_Attribute (*r*s = 0.34 and 0.43, respectively; *p*s < 0.001) and LM_Person (*r*s = 0.51 and 0.65, respectively; *p*s < 0.001), while LM_Debilitating is correlated negatively (*r*s = −0.46 and −0.54, respectively; *p*s < 0.001).

Next, we conducted four hierarchical regressions predicting loneliness, wellbeing, optimism, and self-esteem using the four factors of mindsets as predictors. Prior to running these, we first identified relevant covariates by examining variables associated with any of the mindset predictors and the outcome variables. Specifically, recruitment method (CloudResearch vs. Undergraduates) and personality traits (extraversion, agreeableness, conscientiousness, emotional stability, openness) were included as covariates. In each regression model, these covariates were entered in Step 1, and the four factors (LM_Attribute, LM_Person, LM_Debilitating, LM_Feel) were entered in Step 2.

In the first analysis examining loneliness, the Step 1 model was significant, R^2^ = 0.31, *F*(6, 375) = 27.71, *p* < 0.001. In Step 2, the addition of the loneliness factors explained a significant increase in variance (ΔR^2^ = 0.27, *F* change (4, 371) = 60.47, *p* < 0.001). In this final model, extraversion (β = −0.16, *p* < 0.001) and emotional stability (β = −0.13, *p* = 0.002) were significant predictors of loneliness, as well as LM_Person (β = −0.45, *p* < 0.001) and LM_Debilitating (β = 0.28, *p* < 0.001), indicating that a stronger growth mindset regarding a lonely person was related negatively to loneliness and a stronger debilitating mindset of loneliness was related positively to loneliness (see Table S2 in Supplemental).

In the second analysis examining predictors of wellbeing, the Step 1 model was significant (R^2^ = 0.34, *F* (6, 375) = 31.53, *p* < 0.001). In Step 2, the addition of the mindset factors explained a significant increase in variance (ΔR^2^ = 0.04, *F* change (4, 371) = 5.45, *p* < 0.001). In the final model, extraversion (β = 0.26, *p* < 0.001), conscientiousness (β = 0.16, *p* < 0.001), and emotional stability (β = 0.23, *p* < 0.001) were significant and positive predictors of wellbeing. Among the loneliness mindset variables, LM_Attribute (β = 0.15, *p* = 0.013) was a significant positive predictor, indicating that a stronger growth mindset regarding the attribute of loneliness was related to greater wellbeing (see Table S2 in Supplemental).

In the third analysis examining predictors of optimism, the Step 1 model was significant (R^2^ = 0.45, *F* (6, 375) = 50.91, *p* < 0.001). In Step 2, the addition of each mindset factor explained a significant increase in variance (ΔR^2^ = 0.08, *F* change (4, 371) = 14.95, *p* < 0.001). In the final model, emotional stability (β = 0.34, *p* < 0.001), extraversion (β = 0.19, *p* < 0.001), openness (β = 0.10, *p* = 0.020), and recruitment source (β = 0.08, *p* = 0.045) were significant predictors of optimism. Among the loneliness mindset variables, LM_Attribute (β = 0.12, *p* = 0.024) and LM_Person (β = 0.20, *p* < 0.001) were positively associated with optimism, while LM_Debilitating (β = −0.13, *p* = 0.004) was negatively associated, indicating that a stronger growth mindset regarding the attribute of loneliness and regarding a lonely person is related positively to optimism and a stronger debilitating mindset of loneliness was related negatively to optimism (see Table S2 in Supplemental).

Finally, in the fourth analysis examining predictors of self-esteem, the Step 1 model was significant (R^2^ = 0.54, *F* (6, 375) = 74.10, *p* < 0.001). Adding the loneliness mindset variables in Step 2 significantly improved model fit (ΔR^2^ = 0.15, *F* change (4, 371) = 45.18, *p* < 0.001). In the final model, emotional stability (β = 0.28, *p* < 0.001), extraversion (β = 0.19, *p* < 0.001), and conscientiousness (β = 0.19, *p* < 0.001) emerged as significant positive predictors of self-esteem. Among the loneliness mindset variables, LM_Attribute (β = 0.11, *p* = 0.01) and LM_Person (β = 0.34, *p* < 0.001) were associated positively with self-esteem, while a LM_Debilitating (β = −0.15, *p* < 0.001) was negatively associated (see Table S2 in Supplemental).

## 7. Discussion: Study 2

Study 2 was designed to replicate and extend the findings from Study 1 by confirming the factor structure of the M-LAT, testing additional aspects of construct validity, and expanding predictive validity. First, we successfully confirmed the four-factor structure of the M-LAT using CFA. The model demonstrated acceptable fit, with factor loadings and reliabilities comparable to those found in Study 1. This consistency across independent samples strengthens confidence in the validity of the scale and the conceptual distinctions between the four mindset dimensions: Lonely Attribute (LM_Attribute), Lonely People (LM_Person), Loneliness as Enhancing (LM_Enhancing), and Loneliness as Debilitating (LM_Debilitating). However, the fit indices were adequate, not great, and thus more work is needed to replicate the proposed structure.

Second, Study 2 expanded construct validity by examining links between loneliness mindsets and other well-established mindsets (i.e., mindsets of people and emotions) as well as personality traits. As expected, LM_Attribute, LM_Person, and LM_Enhancing were positively associated with growth-oriented beliefs in related domains, whereas LM_Debilitating showed significant negative associations. These patterns support the idea that loneliness mindsets share core properties with other domain-specific mindsets, while also maintaining discriminant validity, reflecting distinct belief structures. Additionally, the Big Five personality traits were associated with loneliness beliefs, with neuroticism showing the strongest links. This finding is consistent with prior research demonstrating that neuroticism is particularly associated with fixed beliefs (Satchell et al., 2017; Zirenko, 2018). Finally, consistent with Study 1, men reported stronger debilitating loneliness beliefs, providing some evidence of demographic correlates that warrant additional research to understand.

Third, we started to explore the predictive validity of the M-LAT factors in relation to social and psychological wellbeing. Overall, LM_Attribute, LM_Person, and LM_Debilitating emerged as the most robust predictors in the regression models. Interestingly, LM_Enhancing did not relate to any outcomes. LM_Attribute was the only predictor of general wellbeing and was also related positively to optimism and self-esteem. Both LM_Person and LM_Debilitating significantly predicted loneliness, optimism, and self-esteem. Specifically, stronger growth mindsets about lonely people were associated with lower loneliness and higher optimism and self-esteem, whereas stronger debilitating beliefs about loneliness were associated with higher loneliness and lower optimism and self-esteem. These effects are similar to work suggesting that believing that social skills can improve over time can reduce loneliness (Taniguchi, 2018). These findings hold when controlling for recruitment method (CloudResearch vs. Undergraduates) and personality traits (extraversion, agreeableness, conscientiousness, emotional stability, openness). Overall, these results suggest that interventions targeting mindsets of people and debilitating mindsets might have the most impact.

## 8. General Discussion

### 8.1. Overview

We took a tripartite approach to create and test a new measure of three interconnected mindsets about the nature of loneliness with a focus on (a) the changeability of loneliness as an attribute, (b) the nature of people who are lonely, and (c) the experience of loneliness. Findings from both studies support the idea that these are indeed distinct concepts, with four factors emerging—mindsets about the changeable or fixed nature of loneliness, mindsets about the changeable or fixed nature of people who are lonely, as well as enhancing and debilitating mindsets related to the experience of being lonely. Absolute correlations among factors across both studies ranged from 0.00 to 0.52, with the strongest links among the attribute and enhancing factors in Study 2 and the weakest links between person and enhancing across both studies. The correlations among factors overall suggest a clear need to consider each unique factor. We also found that the factors differentially predicted social and psychological wellbeing, contributing to mindset theory and emerging work outlining the importance of differentiating between types of mindsets, especially when considering experiences such as loneliness (e.g., Yeager et al., 2022).

The findings are in line with work that highlights both attribute and person mindsets. For example, in the context of addiction, there were distinct attribute and person mindset factors that differentially related to outcomes (e.g., Burnette et al., 2024). Related, recent work also suggests that enhancing and debilitating mindsets may indeed be distinctive (Caleon et al., 2023; T. T. Chen et al., 2025; Karampas et al., 2020). In the current work, these mindsets are only weakly related to each other, and debilitating mindsets consistently predicted outcomes, whereas enhancing mindsets had weak and inconsistent links. This is in line with related theoretical perspectives that outline the power of the negative to outweigh the positive (e.g., Baumeister et al., 2001). Yet, related work in the context of stress reports these mindsets fall along a single continuum and also consistently predict stress (Crum et al., 2013, 2017). Overall, more work is needed to understand the discrepancies across the contexts and to identify the boundary conditions of enhancing and debilitating mindsets.

### 8.2. Theoretical and Practical Implications

Although our primary focus was on loneliness, we propose that the tripartite theoretical framework applied to assessing loneliness mindsets can be extended to related contexts such as anxiety, emotions and more. For example, people can hold distinct beliefs about the feeling of anxiety, people with anxiety, and the experience of anxiety. Similar to loneliness, anxiety can be evolutionary adaptive (Bateson et al., 2011) and a signal that a different strategy is needed. Indeed, there is a movement to lean into anxiety and understand the potential enhancing benefits (Dennis-Tiwary, 2022; Lee et al., 2006; Nair et al., 2024). We hope that the approach taken here encourages future work to not only distinguish between mindsets across different domains (e.g., loneliness vs. emotions) but also to differentiate between beliefs about attributes, people, and experiences within the same domain.

In terms of practical applications, our findings suggest that cultivating a mindset that frames loneliness as a modifiable and enhancing experience, rather than a fixed personal debilitating flaw, may help to improve individual’s social and psychological wellbeing. For example, the development of our loneliness mindset measure, alongside its initial associations with psychological and social wellbeing, may have important implications for psychotherapeutic approaches such as cognitive-behavioral therapy (CBT), which could target maladaptive beliefs about loneliness to enhance treatment outcomes. Additionally, mindset-focused interventions could consider a synergetic approach that includes messages seeking to foster growth mindsets about the nature of people and those that seek to offset a debilitating mindset. Such an approach may promote resiliency and wellbeing by addressing both how individuals perceive themselves and how they interpret experiences (Jamieson et al., 2023). The integrated mindset model proposed in the current work may outperform single-mindset interventions, especially in high-stress contexts and could be applied to multiple areas where individuals are especially at risk of feeling lonely, such as major life transitions (e.g., becoming a parent, starting college), digital overuse (e.g., social media addiction), and interpersonal struggles (e.g., social anxiety, divorce). Such an approach is in line with more recent synergetic approaches that seek to foster stronger growth mindsets and enhancing mindsets in adolescents and young adults (e.g., Yeager et al., 2022). Future research can use design thinking and randomized “A/B” experiments (Yeager et al., 2016) to inform intervention development, especially those focusing on synergetic approaches to improve wellbeing across clinical and social contexts.

### 8.3. Limitations and Future Research Directions

Before findings are put into practice several limitations warrant consideration and we suggest multiple pathways for future research. Given the correlational design of our studies, causal inferences remain tentative. Future studies should employ experimental manipulations of mindsets, especially those using newer techniques to foster stronger shifts such as a meta-cognitive approach (e.g., Crum et al., 2023). Moreover, this work should also seek to outline the causal pathways through which mindsets might improve wellbeing. For example, do different types of mindsets work through different mediating paths? Might experience mindsets impact wellbeing via reappraisals, whereas mindsets of attributes work via a sense of agency? Additionally, future research should more closely examine the gender difference we observed, with men reporting stronger debilitating mindsets about loneliness than women. This pattern may help explain both the higher rates of loneliness among men and their lower likelihood of reaching out for social support when feeling lonely (Barreto et al., 2021; Goddard & Parker, 2025). Additionally, we only examined social anxiety, but depression is also closely tied to loneliness with reciprocal influences on wellbeing (e.g., J. T. Cacioppo et al., 2006). Thus, future work should include additional assessments of social and psychological wellbeing to explore links to each factor of mindsets.

From a methodological standpoint, additional work is needed that replicates findings. For example, confirmatory studies with preregistered hypotheses are needed. Analyses such as parallel factor analysis could also help solidify the underlying structure of the loneliness mindset measure. Additionally, our participant samples were primarily composed of WEIRD (Western, Educated, Industrialized, Rich, and Democratic) populations (Henrich et al., 2010). Cultural norms significantly influence how loneliness is experienced and addressed. For example, cultures emphasizing collective harmony may discourage open discussion of loneliness, potentially dampening the effectiveness of a mindset approach (e.g., Kim & Markus, 1999). Understanding these cultural differences is essential for developing globally relevant interventions. Finally, while the present study relied on self-reported social and psychological wellbeing, future work should incorporate behavioral and physiological indicators, such as observed social engagement, cortisol reactivity, or vagal tone (Doane & Adam, 2010; Piejka et al., 2021; Queen et al., 2014).

### 8.4. Conclusions

In summary, we introduced a novel framework for understanding loneliness mindsets. By integrating theoretical insights from varied approaches to mindsets, including attribute, people, and experiences, we developed a comprehensive measure. Preliminary evidence supports the measure’s validity and finds many of these mindset factors predict social and psychological wellbeing. We hope that this foundational work will guide future psychometric work seeking to develop new mindset measures as well as research that develops synergetic interventions aimed at fostering adaptive coping strategies and reducing the negative impact of loneliness. The loneliness epidemic continues to grow at great cost to both individuals, institutions, and societies (Buecker et al., 2021; S. Cacioppo et al., 2015; Meisters et al., 2021). We provide, in the present research, a compelling, theory-driven, foundation from which future work can seek to address the loneliness epidemic—one that should be combined with context conscious and system-level approaches (Crowe et al., 2024; Holt-Lunstad, 2021). We hope that our mindset-based approach instigates multiple impactful future studies to combat the costs of loneliness on wellbeing.

## Figures and Tables

**Table 1:** Communalities and factor loadings (Studies 1 and 2).

Constructs	EFA (Study 1)		CFA (Study 2)
	Communalities	Rotated Factor Loadings	Standardized Factor Loadings
**Lonely Attribute Mindset (LM_Attribute)**			
I see loneliness as a temporary state.	0.54	0.79	0.76
My loneliness is temporary.	0.65	0.80	0.79
If I feel lonely, that is likely to change over time.	0.66	0.76	0.73
Loneliness is something that is likely to change over time.	0.71	0.79	0.75
**Lonely People Mindset (LM_Person)**			
Being lonely is an inherent part of who I am.	0.67	0.90	0.85
Loneliness is part of my personality.	0.76	0.90	0.78
I have come to accept that being a lonely person is a fixed aspect of who I am.	0.78	0.78	0.80
I cannot do anything to change my loneliness.	0.81	0.89	0.75
I will always be lonely and there isn’t much I can do to change that.	0.84	0.88	0.85
I will always be lonely.	0.75	0.88	0.87
**Loneliness as Enhancing Mindset (LM_Enhancing)**			
If I lean into loneliness, I can grow from the experience.	0.49	0.72	0.75
When I experience loneliness, I think it can help me grow.	0.73	0.88	0.86
Loneliness is part of life and thus provides a lesson.	0.62	0.73	0.62
**Loneliness as Debilitating Mindset (LM_Debilitating)**			
Loneliness makes me feel unmotivated.	0.65	0.82	0.75
Loneliness is debilitating.	0.77	0.90	0.73
Once I start to experience loneliness, I feel trapped.	0.56	0.60	0.80

**Table 2:** M-LAT subscale reliabilities, means, standard deviations, and correlations.

**Study 1**	**Reliability**	**M (SD)**	**Range**	**LM_Attribute**	**LM_Person**	**LM_Enhancing**	**LM_Debilitating**
LM_Attribute	0.90	5.25 (1.01)	1.0–7.0	—			
LM_Person	0.87	5.48 (1.13)	1.0–7.0	0.42 ***	—		
LM_Enhancing	0.80	4.68 (1.20)	1.0–7.0	0.31 ***	−0.004	—	
LM_Debilitating	0.80	4.67 (1.42)	1.0–7.0	0.18 **	−0.08	−0.07	—
**Study 2**	**Reliability**	**M (SD)**	**Range**	**LM_Attribute**	**LM_Person**	**LM_Enhancing**	**LM_Debilitating**
LM_Attribute	0.84	5.12 (1.15)	1.0–7.0	—			
LM_Person	0.92	5.23 (1.40)	1.0–7.0	0.42 ***	—		
LM_Enhancing	0.78	4.38 (1.29)	1.0–7.0	0.52 ***	−0.07	—	
LM_Debilitating	0.80	4.22 (1.48)	1.0–7.0	−0.12 *	−0.50 ***	0.10 *	—

Note. LM_Attribute = Lonely Attribute Mindset, LM_Person = Lonely People Mindset, LM_Enhancing = Loneliness as Enhancing Mindset, LM_Debilitating = Loneliness as Debilitating Mindset, LM_Attribute, and LM_Person are coded such that higher scores indicate a stronger growth mindset. LM_Enhancing and LM_Debilitating are coded such that higher scores indicate stronger beliefs that loneliness is enhancing or debilitating, respectively; * *p* < 0.05. ** *p* < 0.01. *** *p* < 0.001.

**Table 3:** Descriptive statistics, reliabilities, and correlations for Study 1 variables.

Measure	M	SD	1	2	3	4	5	6	7	8	9	10	11	12
1. LM_Attribute	5.26	1.01	—											
2. LM_Person	5.48	1.13	0.42 ***	—										
3. LM_Enhancing	4.68	1.20	0.31 ***	−0.004	—									
4. LM_Debilitating	4.67	1.42	0.18 **	−0.08	−0.07	—								
5. Mindset of Intelligence	5.26	1.13	0.18 **	0.26 ***	0.20 **	−0.07	—							
6. Mindset of Shyness	4.53	1.04	−0.01	0.36 ***	−0.02	0.15 *	0.20 **	—						
7. Loneliness	3.42	1.47	−0.04	−0.29 ***	−0.001	0.31 ***	0.03	0.08	—					
8. Lonely Person	0 = No 1 = Yes	—	−0.17 **	−0.34 ***	−0.01	0.21 **	0.05	0.11	0.50 ***	—				
9. Social Anxiety	3.17	1.07	0.10	−0.29 ***	0.13 *	0.26 ***	0.02	0.34 ***	0.36 ***	0.29 ***	—			
10. Age	19.30	1.39	0.05	0.002	−0.06	0.06	−0.04	−0.11	0.09	0.20 **	−0.06	—		
11. School Year	1.73	1.00	0.09	0.06	0.11	0.03	0.03	−0.13 *	0.01	0.15 *	−0.06	0.79 **	—	
12. Gender	—	—	0.10	0.04	0.02	0.21 ***	0.12	−0.13 *	0.21 ***	0.08	0.21 **	−0.04	0.03	—
13. PSES	4.76	1.51	−0.10	−0.10	0.07	−0.01	−0.05	−0.03	0.06	0.09	0.04	0.02	−0.02	−0.002

Note. LM_Attribute = Lonely Attribute Mindset, LM_Person = Lonely People Mindset; LM_Enhancing = Loneliness as Enhancing Mindset, LM_Debilitating = Loneliness as Debilitating Mindset, LM_Attribute and LM_Person are coded such that higher scores indicate a stronger growth mindset. LM_Enhancing and LM_Debilitating are coded such that higher scores indicate stronger beliefs that loneliness is enhancing or debilitating, respectively; gender coded such that woman = 0 and man = 1 and non-binary participants excluded; PSES = perceived socioeconomic status; * *p* < 0.05. ** *p* < 0.01. *** *p* < 0.001.

**Table 4:** Descriptive statistics, reliabilities, and correlations for Study 2 variables.

Measure	M	SD	1	2	3	4	5	6	7	8	9	10	11	12	13	14	15	16	17
1. LM_Attribute	5.12	1.15	—																
2. LM_Person	5.23	1.40	0.42 ***	—															
3. LM_Enhancing	4.38	1.29	0.52 ***	−0.07	—														
4. LM_Debilitating	4.22	1.48	−0.12 *	−0.50 ***	0.10 *	—													
5. Mindset of People	4.54	1.05	0.22 ***	0.22 ***	0.10 *	−0.17 ***	—												
6. Mindset of Emotion	4.77	1.20	0.34 ***	0.45 ***	0.14 **	−0.44 ***	0.29 ***	—											
7. Extra.	3.42	1.50	0.17 ***	0.29 ***	0.02	−0.14 *	0.04	0.15 **	—										
8. Agree.	4.99	1.14	0.17 **	0.26 ***	−0.01	−0.15 *	0.18 ***	0.21 ***	0.08	—									
9. Conscient.	5.14	1.24	0.14 **	0.28 ***	0.01	−0.33 ***	−0.01	0.25 ***	0.18 ***	0.28 ***	—								
10. Emot. Stab.	4.31	1.44	0.27 ***	0.33 ***	0.04	−0.48 ***	0.10 *	0.52 ***	0.17 ***	0.36 ***	0.43 ***	—							
11. Open.	5.17	1.10	0.19 ***	0.33 ***	0.04	−0.17 ***	0.14 **	0.21 ***	0.31 ***	0.31 ***	0.19 ***	0.23 ***	—						
12. Loneliness	41.60	14.88	−0.26 ***	−0.67 ***	0.07	0.60 ***	−0.13 **	−0.47 ***	−0.33 ***	−0.22 ***	−0.34 ***	−0.45 ***	−0.23 ***	—					
13. Wellbeing	4.34	4.35	0.29 ***	0.39 ***	0.01	−0.36 ***	0.01	0.32 ***	0.38 ***	0.21 ***	0.38 ***	0.45 ***	0.19 ***	−58 ***	—				
14. Self-Esteem	4.67	1.20	0.43 ***	0.65 ***	0.08	−0.54 ***	0.16 **	0.55 ***	0.41 ***	0.30 ***	0.50 ***	0.61 ***	0.36 ***	−0.72 ***	0.69 ***	—			
15. Optimism	4.37	1.19	0.34 ***	0.51 ***	0.02	−0.46 ***	0.16 ***	0.52 ***	0.36 ***	0.32 ***	0.34 ***	0.58 ***	0.37 ***	−0.59 ***	0.57 ***	0.74 ***	—		
16. Age	21.06	0.61	−0.18 ***	−0.30 ***	−0.02	0.04	−0.05	−0.03	−0.18 ***	0.16 **	−0.05	0.04	0.05	0.16 **	−0.15 **	−0.12 *	−0.02	—	
17. Gender	—	—	−0.06	−0.05	−0.002	−0.13 *	0.05	0.16 **	0.02	0.05	0.05	0.31 ***	-0.03	-0.05	0.06	−0.13 *	−0.11 *	−0.02	—
18. PSES	5.37	1.69	−0.19 ***	−0.23 ***	−0.06	0.11 *	−0.05	−0.19 ***	−0.13 *	−0.09	−0.11 *	−0.17 ***	−0.03	0.19 ***	−0.20 ***	−0.21 ***	−0.18 ***	0.29 ***	0.06

Note. LM_Attribute = Lonely Attribute Mindset; LM_Person = Lonely People Mindset; LM_Enhancing = Loneliness as Enhancing Mindset; LM_Debilitating = Loneliness as Debilitating Mindset; LM_Attribute and LM_Person are coded such that higher scores indicate a stronger growth mindset. LM_Enhancing and LM_Debilitating are coded such that higher scores indicate stronger beliefs that loneliness is enhancing or debilitating, respectively; Gender coded such that woman = 0 and man = 1 and non-binary participants excluded; PSES = Perceived socioeconomic status; * *p* < 0.05. ** *p* < 0.01. *** *p* < 0.001.

## Data Availability

Data are available on OSF. (https://osf.io/y6wcf/?view_only=af19aa5812944e4497b6d7df9762413c, accessed on 2 June 2025).

## Notes

1 We also assessed open-ended questions about loneliness that we did not code or include in analyses.

2 We also included a single item about the inclination to conceal loneliness as a potential internalization of stigma item for other ongoing work.

## Supplementary Materials

The following supporting information can be downloaded at: https://www.mdpi.com/article/10.3390/bs15091196/s1, Figure S1: Scree Plot; Table S1: Sociodemographic Characteristics of Participants in Studies 1 and 2; Table S2: Hierarchical Regressions in Study 2.

## References

[B1-behavsci-15-01196] Akaike H. (1974). A new look at the statistical model identification. IEEE Transactions on Automatic Control.

[B2-behavsci-15-01196] American Psychiatric Association (2025). Media advisory: New polling data on loneliness, experts available from American Psychiatric Association.

[B3-behavsci-15-01196] Barreto M., Victor C., Hammond C., Eccles A., Richins M. T., Qualter P. (2021). Loneliness around the world: Age, gender, and cultural differences in loneliness. Personality and Individual Differences.

[B4-behavsci-15-01196] Bateson M., Brilot B., Nettle D. (2011). Anxiety: An evolutionary approach. The Canadian Journal of Psychiatry.

[B5-behavsci-15-01196] Baumeister R. F., Bratslavsky E., Finkenauer C., Vohs K. D. (2001). Bad is stronger than good. Review of General Psychology.

[B6-behavsci-15-01196] Beavers A. S., Lounsbury J. W., Richards J. K., Huck S. W., Skolits G. J., Esquivel S. L. (2013). Practical considerations for using exploratory factor analysis in educational research. Practical Assessment, Research & Evaluation.

[B7-behavsci-15-01196] Becker W., Burnette J. L., Hoyt C. L. (2023). Coping in the time of COVID-19: Mindsets and the stories we tell. Journal of Applied Social Psychology.

[B8-behavsci-15-01196] Bentler P. M. (1990). Comparative fit indexes in structural models. Psychological Bulletin.

[B9-behavsci-15-01196] Buecker S., Maes M., Denissen J. J. A. (2020). Loneliness and the big five personality traits: A meta–analysis. European Journal of Personality.

[B10-behavsci-15-01196] Buecker S., Mund M., Chwastek S., Sostmann M., Luhmann M. (2021). Is loneliness in emerging adults increasing over time? A preregistered cross-temporal meta-analysis and systematic review. Psychological Bulletin.

[B11-behavsci-15-01196] Burch K. (n.d.). Feeling lonely in college.

[B12-behavsci-15-01196] Burnette J. L., Hoyt C. L., Becker W., O’Keefe P. A. (2024). Addiction stigma: Potential costs and benefits of beliefs about the nature of addiction. Stigma and Health.

[B13-behavsci-15-01196] Burnette J. L., Knouse L. E., Vavra D. T., O’Boyle E., Brooks M. A. (2020). Growth mindsets and psychological distress: A meta-analysis. Clinical Psychology Review.

[B14-behavsci-15-01196] Burnette J. L., O’Boyle E. H., VanEpps E. M., Pollack J. M., Finkel E. J. (2013). Mind-sets matter: A meta-analytic review of implicit theories and self-regulation. Psychological Bulletin.

[B15-behavsci-15-01196] Byrne B. M. (2001). Structural equation modeling with AMOS, EQS, and LISREL: Comparative approaches to testing for the factorial validity of a measuring instrument. International Journal of Testing.

[B16-behavsci-15-01196] Cacioppo J. T., Cacioppo S. (2018). The growing problem of loneliness. The Lancet.

[B17-behavsci-15-01196] Cacioppo J. T., Hawkley L. C. (2009). Perceived social isolation and cognition. Trends in Cognitive Sciences.

[B18-behavsci-15-01196] Cacioppo J. T., Hughes M. E., Waite L. J., Hawkley L. C., Thisted R. A. (2006). Loneliness as a specific risk factor for depressive symptoms: Cross-sectional and longitudinal analyses. Psychology and Aging.

[B19-behavsci-15-01196] Cacioppo S., Grippo A. J., London S., Goossens L., Cacioppo J. T. (2015). Loneliness: Clinical import and interventions. Perspectives on Psychological Science.

[B20-behavsci-15-01196] Caleon I. S., Kadir M. B. S., Tan C. S., Chua J., Ilham N. Q. B. (2023). Stress mindset, coping strategies, and well-being of secondary students in Singapore during the COVID-19 pandemic. Educational Psychology.

[B21-behavsci-15-01196] Carpenter S. (2018). Ten steps in scale development and reporting: A guide for researchers. Communication Methods and Measures.

[B22-behavsci-15-01196] Chen L., Yang F. (2022). Social support and loneliness among Chinese rural-to-urban migrant children: A moderated mediation analysis of the roles of social competence and stress mindset. International Journal of Environmental Research and Public Health.

[B23-behavsci-15-01196] Chen T. T., Ching B. H. H., Wu H. X., Li X. Y. (2025). Exploring a two-factor structure of stress mindsets in academic contexts: Their connections with emotional and behavioral outcomes. Social Psychology of Education.

[B24-behavsci-15-01196] Chiu C. Y., Hong Y. Y., Dweck C. S. (1997). Lay dispositionism and implicit theories of personality. Journal of Personality and Social Psychology.

[B25-behavsci-15-01196] Connor K. M., Kobak K. A., Churchill L. E., Katzelnick D., Davidson J. R. (2001). Mini-SPIN: A brief screening assessment for generalized social anxiety disorder. Depression and Anxiety.

[B26-behavsci-15-01196] Couto S. D. (2023). Loneliness is now a “global public health concern,” says who—National. Global News.

[B27-behavsci-15-01196] Crowe C. L., Liu L., Bagnarol N., Fried L. P. (2024). Loneliness prevention and the role of the public health system. Perspectives in Public Health.

[B28-behavsci-15-01196] Crum A. J., Akinola M., Martin A., Fath S. (2017). The role of stress mindset in shaping cognitive, emotional, and physiological responses to challenging and threatening stress. Anxiety, Stress, & Coping.

[B29-behavsci-15-01196] Crum A. J., Salovey P., Achor S. (2013). Rethinking stress: The role of mindsets in determining the stress response. Journal of Personality and Social Psychology.

[B30-behavsci-15-01196] Crum A. J., Santoro E., Handley-Miner I., Smith E. N., Evans K., Moraveji N., Achor S., Salovey P. (2023). Evaluation of the “rethink stress” mindset intervention: A metacognitive approach to changing mindsets. Journal of Experimental Psychology: General.

[B31-behavsci-15-01196] Degol J. L., Wang M. T., Zhang Y., Allerton J. (2018). Do growth mindsets in math benefit females? Identifying pathways between gender, mindset, and motivation. Journal of Youth and Adolescence.

[B32-behavsci-15-01196] Dennis-Tiwary T. (2022). Future tense: Why anxiety is good for you (Even though it feels bad).

[B33-behavsci-15-01196] Destin M., Hanselman P., Buontempo J., Tipton E., Yeager D. S. (2019). Do student mindsets differ by socioeconomic status and explain disparities in academic achievement in the United States?. AERA Open.

[B34-behavsci-15-01196] Diener E. D., Emmons R. A., Larsen R. J., Griffin S. (1985). The satisfaction with life scale. Journal of Personality Assessment.

[B35-behavsci-15-01196] Doane L. D., Adam E. K. (2010). Loneliness and cortisol: Momentary, day-to-day, and trait associations. Psychoneuroendocrinology.

[B36-behavsci-15-01196] Douglas B. D., Ewell P. J., Brauer M. (2023). Data quality in online human-subjects research: Comparisons between MTurk, Prolific, CloudResearch, Qualtrics, and SONA. PLoS ONE.

[B37-behavsci-15-01196] Dweck C. S. (1999). Self-theories: Their role in motivation, personality, and development.

[B38-behavsci-15-01196] Dweck C. S. (2006). Mindset: The new psychology of success.

[B39-behavsci-15-01196] Dweck C. S., Leggett E. L. (1988). A social-cognitive approach to motivation and personality. Psychological Review.

[B40-behavsci-15-01196] Eccleston C., Crombez G. (1999). Pain demands attention: A cognitive–affective model of the interruptive function of pain. Psychological Bulletin.

[B41-behavsci-15-01196] Fabrigar L. R., Wegener D. T., MacCallum R. C., Strahan E. J. (1999). Evaluating the use of exploratory factor analysis in psychological research. Psychological Methods.

[B42-behavsci-15-01196] Goddard I., Parker K. (2025). Men, women and social connections.

[B43-behavsci-15-01196] Goretzko D., Pham T. T. H., Bühner M. (2021). Exploratory factor analysis: Current use, methodological developments and recommendations for good practice. Current Psychology.

[B44-behavsci-15-01196] Gorsuch R. L. (2014). Factor analysis: Classic edition.

[B45-behavsci-15-01196] Gosling S. D., Rentfrow P. J., Swann W. B. (2003). A very brief measure of the Big-Five personality domains. Journal of Research in Personality.

[B46-behavsci-15-01196] Gow A. J., Pattie A., Whiteman M. C., Whalley L. J., Deary I. J. (2007). Social support and successful aging: Investigating the relationships between lifetime cognitive change and life satisfaction. Journal of Individual Differences.

[B47-behavsci-15-01196] Hartman R., Moss A. J., Jaffe S. N., Rosenzweig C., Litman L., Robinson J. (2023). Introducing connect by CloudResearch: Advancing online participant recruitment in the digital age.

[B48-behavsci-15-01196] Haslam N., Rothschild L., Ernst D. (2000). Essentialist beliefs about social categories. British Journal of Social Psychology.

[B50-behavsci-15-01196] Hawkley L. (2019). Solitary confinement: Effects, practices, and pathways toward reform.

[B51-behavsci-15-01196] Hawkley L. C. (2022). Loneliness and health. Nature Reviews Disease Primers.

[B52-behavsci-15-01196] Hawkley L. C., Cacioppo J. T. (2010). Loneliness matters: A theoretical and empirical review of consequences and mechanisms. Annals of Behavioral Medicine.

[B49-behavsci-15-01196] Henrich J., Heine S. J., Norenzayan A. (2010). The weirdest people in the world?. Behavioral and Brain Sciences.

[B53-behavsci-15-01196] Henson R. K., Roberts J. K. (2006). Use of exploratory factor analysis in published research: Common errors and some comment on improved practice. Educational and Psychological Measurement.

[B54-behavsci-15-01196] Holt-Lunstad J. (2021). Loneliness and social isolation as risk factors: The power of social connection in prevention. American Journal of Lifestyle Medicine.

[B55-behavsci-15-01196] Hoyt C. L., Burnette J. L. (2025). How mindsets can mitigate or sustain prejudice. Current Directions in Psychological Science.

[B56-behavsci-15-01196] Hoyt C. L., Burnette J. L., Moore A. B. (2024). Trait and social essentialism: Implications for partisan prejudice. Personality and Social Psychology Bulletin.

[B57-behavsci-15-01196] Hu L., Bentler P. M. (1999). Cutoff criteria for fit indexes in covariance structure analysis: Conventional criteria versus new alternatives. Structural Equation Modeling: A Multidisciplinary Journal.

[B58-behavsci-15-01196] Huebschmann N. A., Sheets E. S. (2020). The right mindset: Stress mindset moderates the association between perceived stress and depressive symptoms. Anxiety, Stress, & Coping.

[B59-behavsci-15-01196] Jamieson J. P., Hangen E. J., Lee H. Y., Yeager D. S. (2023). A synergistic mindsets intervention protects adolescents from stress. Nature.

[B60-behavsci-15-01196] John T. (2018). Meet Tracey Crouch, Britain’s minister for loneliness. Time.

[B61-behavsci-15-01196] Kaiser H. F. (1960). The application of electronic computers to factor analysis. Educational and Psychological Measurement.

[B62-behavsci-15-01196] Karaman M. A., Nelson K. M., Cavazos Vela J. (2018). Growth mindset, grit, and optimism as predictors of well-being: A positive psychology perspective. Journal of Counseling & Development.

[B63-behavsci-15-01196] Karampas K., Pezirkianidis C., Stalikas A. (2020). Psychometric properties of the Stress Mindset Measure (SMM) in a Greek sample. Psychology.

[B64-behavsci-15-01196] Kim H., Markus H. R. (1999). Deviance or uniqueness, harmony or conformity? A cultural analysis. Journal of Personality and Social Psychology.

[B65-behavsci-15-01196] Kneeland E. T., Simpson L. E. (2022). Emotion malleability beliefs influence emotion regulation and emotion recovery among individuals with depressive symptoms. Cognition and Emotion.

[B66-behavsci-15-01196] Knowles M. L., Lucas G. M., Baumeister R. F., Gardner W. L. (2015). Choking under social pressure: Social monitoring among the lonely. Personality and Social Psychology Bulletin.

[B67-behavsci-15-01196] Laursen B., Hartl A. C. (2013). Understanding loneliness during adolescence: Developmental changes that increase the risk of perceived social isolation. Journal of Adolescence.

[B68-behavsci-15-01196] Lee W. E., Wadsworth M. E. J., Hotopf M. (2006). The protective role of trait anxiety: A longitudinal cohort study. Psychological Medicine.

[B69-behavsci-15-01196] Levy S. R., Stroessner S. J., Dweck C. S. (1998). Stereotype formation and endorsement: The role of implicit theories. Journal of Personality and Social Psychology.

[B70-behavsci-15-01196] Lim M. H., Rodebaugh T. L., Zyphur M. J., Gleeson J. F. (2016). Loneliness over time: The crucial role of social anxiety. Journal of Abnormal Psychology.

[B71-behavsci-15-01196] Long C. R., Averill J. R. (2003). Solitude: An exploration of benefits of being alone. Journal for the Theory of Social Behaviour.

[B72-behavsci-15-01196] Luhmann M., Buecker S., Rüsberg M. (2023). Loneliness across time and space. Nature Reviews Psychology.

[B73-behavsci-15-01196] McCroskey J. C., Young T. J. (1979). The use and abuse of factor analysis in communication research. Human Communication Research.

[B74-behavsci-15-01196] Meisters R., Westra D., Putrik P., Bosma H., Ruwaard D., Jansen M. (2021). Does loneliness have a cost? A population-wide study of the association between loneliness and healthcare expenditure. International Journal of Public Health.

[B75-behavsci-15-01196] Mertler C. A., Vannatta R. A. (2002). Advanced and multivariate statistical methods: Practical application and interpretation.

[B76-behavsci-15-01196] Molden D. C., Dweck C. S. (2006). Finding “meaning” in psychology: A lay theories approach to self-regulation, social perception, and social development. American Psychologist.

[B77-behavsci-15-01196] Murphy B. A., Costello T. H., Watts A. L., Cheong Y. F., Berg J. M., Lilienfeld S. O. (2020). Strengths and weaknesses of two empathy measures: A comparison of the measurement precision, construct validity, and incremental validity of two multidimensional indices. Assessment.

[B78-behavsci-15-01196] Nair T. K., Waslin S. M., Rodrigues G. A., Datta S., Moore M. T., Brumariu L. E. (2024). A meta-analytic review of the relations between anxiety and empathy. Journal of Anxiety Disorders.

[B79-behavsci-15-01196] Nunnally J. C., Bernstein I. H. (1994). Psychometric Theory.

[B80-behavsci-15-01196] Park C., Majeed A., Gill H., Tamura J., Ho R. C., Mansur R. B., Nasri F., Lee Y., Rosenblat J. D., McIntyre R. S. (2020). The effect of loneliness on distinct health outcomes: A comprehensive review and meta-analysis. Psychiatry Research.

[B81-behavsci-15-01196] Park D., Yu A., Metz S. E., Tsukayama E., Crum A. J., Duckworth A. L. (2018). Beliefs about stress attenuate the relation among adverse life events, perceived distress, and self-control. Child Development.

[B82-behavsci-15-01196] Peer E., Rothschild D., Gordon A., Evernden Z., Damer E. (2022). Data quality of platforms and panels for online behavioral research. Behavior research methods.

[B83-behavsci-15-01196] Piejka A., Wiśniewska M., Thayer J. F., Okruszek Ł. (2021). Brief induction of loneliness decreases vagal regulation during social information processing. International Journal of Psychophysiology.

[B84-behavsci-15-01196] Putnam R. D. (2000). Bowling alone: The collapse and revival of American community.

[B85-behavsci-15-01196] Queen T. L., Stawski R. S., Ryan L. H., Smith J. (2014). Loneliness in a day: Activity engagement, time alone, and experienced emotions. Psychology and Aging.

[B86-behavsci-15-01196] Rhodes M., Leslie S. J., Tworek C. M. (2012). Cultural transmission of social essentialism. Proceedings of the National Academy of Sciences.

[B87-behavsci-15-01196] Rosenberg M. (1965). Rosenberg self-esteem scale (RSE). Acceptance and commitment therapy. Measures Package.

[B88-behavsci-15-01196] Russell D., Peplau L. A., Ferguson M. L. (1978). Developing a measure of loneliness. Journal of Personality Assessment.

[B89-behavsci-15-01196] Ryazanov A. A., Christenfeld N. J. (2018). The strategic value of essentialism. Social and Personality Psychology Compass.

[B90-behavsci-15-01196] Ryff C. D. (1989). Happiness is everything, or is it? Explorations on the meaning of psychological well-being. Journal of Personality and Social Psychology.

[B91-behavsci-15-01196] Sakaluk J. K., Short S. D. (2017). A methodological review of exploratory factor analysis in sexuality research: Used practices, best practices, and data analysis resources. Journal of Sex Research.

[B92-behavsci-15-01196] Satchell L., Hoskins S., Corr P., Moore R. (2017). Ruminating on the nature of intelligence: Personality predicts implicit theories and educational persistence. Personality and Individual Differences.

[B93-behavsci-15-01196] Scheier M. F., Carver C. S., Bridges M. W. (1994). Distinguishing optimism from neuroticism (and trait anxiety, self-mastery, and self-esteem): A reevaluation of the Life Orientation Test. Journal of Personality and Social Psychology.

[B94-behavsci-15-01196] Schell V., De France K., Lin L., Hollenstein T. (2023). The role of avoidance in understanding emotional dysfunction associated with a fixed emotion mindset. Personality and Individual Differences.

[B95-behavsci-15-01196] Schroder H. S., Callahan C. P., Gornik A. E., Moser J. S. (2019). The fixed mindset of anxiety predicts future distress: A longitudinal study. Behavior Therapy.

[B96-behavsci-15-01196] Schroder H. S., Dawood S., Yalch M. M., Donnellan M. B., Moser J. S. (2016). Evaluating the domain specificity of mental health–related mind-sets. Social Psychological and Personality Science.

[B97-behavsci-15-01196] Schwarz G. (1978). Estimating the dimension of a model. The Annals of Statistics.

[B98-behavsci-15-01196] Seligman M. E. P. (2002). Authentic happiness: Using the new positive psychology to realize your potential for lasting fulfillment.

[B99-behavsci-15-01196] Sisk V. F., Burgoyne A. P., Sun J., Butler J. L., Macnamara B. N. (2018). To what extent and under which circumstances are growth mind-sets important to academic achievement? Two meta-analyses. Psychological Science.

[B100-behavsci-15-01196] Snape D., Manclossi S. (2018). Children’s and young people’s experiences of loneliness: 2018.

[B101-behavsci-15-01196] Steiger J. H. (1998). A note on multiple sample extensions of the RMSEA fit index. Structural Equation Modeling: A Multidisciplinary Journal.

[B102-behavsci-15-01196] Tamir M., John O. P., Srivastava S., Gross J. J. (2007). Implicit theories of emotion: Affective and social outcomes across a major life transition. Journal of Personality and Social Psychology.

[B103-behavsci-15-01196] Taniguchi E. (2018). Loneliness and inducing incremental theories of social interactions to produce adaptive change. Personal Relationships.

[B104-behavsci-15-01196] Tao V. Y., Li Y., Wu A. M. (2022). Do not despise failures: Students’ failure mindset, perception of parents’ failure mindset, and implicit theory of intelligence. European Journal of Psychology of Education.

[B105-behavsci-15-01196] Tucker L. R., Lewis C. (1973). A reliability coefficient for maximum likelihood factor analysis. Psychometrika.

[B106-behavsci-15-01196] Tugade M. M., Fredrickson B. L. (2004). Resilient individuals use positive emotions to bounce back from negative emotional experiences. Journal of Personality and Social Psychology.

[B107-behavsci-15-01196] Valentiner D. P., Mounts N. S., Durik A. M., Gier-Lonsway S. L. (2011). Shyness mindset: Applying mindset theory to the domain of inhibited social behavior. Personality and Individual Differences.

[B108-behavsci-15-01196] Wang F., Gao Y., Han Z., Yue Y., Long Z., Jiang X., Wu Y., Pei B., Cao Y., Ye J., Wang M., Zhao Y. (2023). A systematic review and meta-analysis of 90 cohort studies of social isolation, loneliness and mortality. Nature Human Behaviour.

[B109-behavsci-15-01196] Yeager D. S., Bryan C. J., Gross J. J., Murray J. S., Krettek Cobb D., Santos P. H. F., Gravelding H., Johnson M., Jamieson J. P. (2022). A synergistic mindsets intervention protects adolescents from stress. Nature.

[B110-behavsci-15-01196] Yeager D. S., Romero C., Paunesku D., Hulleman C. S., Schneider B., Hinojosa C. P., Lee H. Y., O’Brien J., Flint K., Roberts A., Trott J., Greene D., Walton G. M., Dweck C. S. (2016). Using design thinking to improve psychological interventions: The case of the growth mindset during the transition to high school. Journal of Educational Psychology.

[B111-behavsci-15-01196] Zirenko M. S. (2018). Implicit theories of intelligence and personality: Relations to intelligence, motivation and personality. Journal of Higher School of Economics.

