# Supplementary material for: Loneliness Mindsets: A New Measurement Approach and Implications for Predicting Wellbeing
Source: Behav Sci (Basel). 2025 Sep 2;15(9):1196. doi: 10.3390/bs15091196 (PMC12466599; doi:10.3390/bs15091196)
Supplement: Supplementary file 1 [file behavsci-15-01196-s001.zip › behavsci-3720078-supplementary.pdf]

## Supplemental Figures and Tables

**Figure S1.** Scree plot.

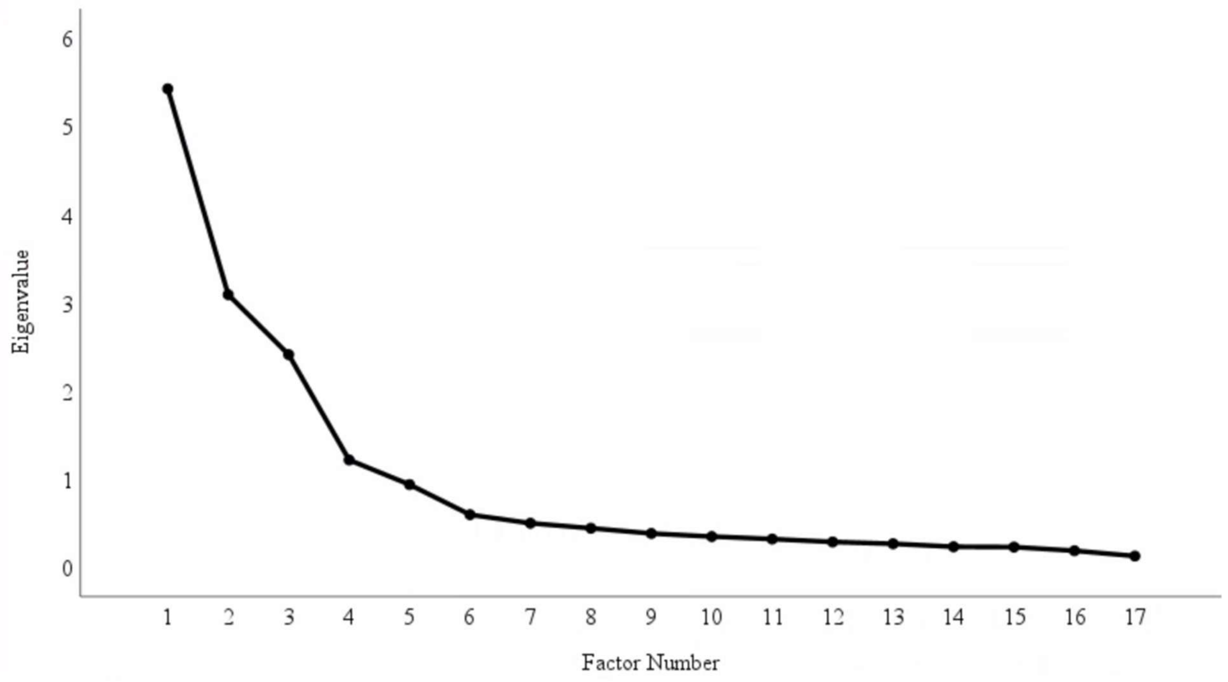

**Table S1.** Sociodemographic characteristics of participants in Studies 1 and 2.

| Participant Characteristic | Study 1 (N = 243) | Study 2 (n = 382) |
|---|---|---|
| Gender | | |
| Woman | 119, 49.2% | 174, 45.5% |
| Man | 120, 49.6% | 195, 51% |
| Non-binary | 2, .8% | 11, 2.9% |
| Prefer not to say | 1, .4% | 2, .6% |
| Race / Ethnicity | | |
| Black or African American | 34 | 110 |
| White | 166 | 216 |
| American Indian/Alaskan Native | 2 | 4 |
| Asian | 32 | 41 |
| Native Hawaiian or other Pacific Islander | 0 | 3 |
| Hispanic/Latino/ Spanish | 24 | 42 |
| Not specified | 0 | 1 |
| Prefer not to say | 2 | 1 |
| Age | 19.30 (1.39) | 21.06 (2.30) |
| School Year | | |
| Year 1 | 135, 55.8% | — |
| Year 2 | 61, 25.2% | — |
| Year 3 | 27, 11.2% | — |
| Year 4 | 15, 6.2% | — |
| Year 5+ | 4, 1.6% | — |
| PSES | 4.76 (1.51) | 5.37 (1.69) |

*Note.* Categorical variables displayed; No, % and continuous variables are shown as mean (SD); Participants were asked to report all race/ethnicity options that apply; PSES = perceived socioeconomic status.

**Table S2.** Hierarchical regressions in Study 2

| Outcome | Step | $R^2$ | $\Delta R^2$ | F for Step / ΔF | Significant Predictors (Final Model) |
|---|---|---|---|---|---|
| Loneliness | | | | | |
| | 1 | .31 | — | F(6, 375) = 27.71, *p* < .001 | Extraversion (β = –.16, *p* < .001)<br>Emotional Stability (β = –.13, *p* = .002) |
| | 2 | .58 | .27 | F(4, 371) = 60.47, *p* < .001 | LM_Person (β = –.45, *p* < .001)<br>LM_Debilitating (β = .28, *p* < .001) |
| Wellbeing | | | | | |
| | 1 | .34 | — | F(6, 375) = 31.53, *p* < .001 | Extraversion (β = .26, *p* < .001)<br>Conscientiousness (β = .16, *p* < .001)<br>Emotional Stability (β = .23, *p* < .001) |
| | 2 | .37 | .04 | F(4, 371) = 5.45, *p* < .001 | LM_Attribute (β = .15, *p* = .013)<br>LM_Debilitating (β = –.10, *p* = .055)† |
| Optimism | | | | | |
| | 1 | .45 | — | F(6, 375) = 50.91, *p* < .001 | Emotional Stability (β = .34, *p* < .001)<br>Extraversion (β = .19, *p* < .001)<br>Openness (β = .10, *p* = .020)<br>Sample Source (β = .08, *p* = .045) |
| | 2 | .53 | .08 | F(4, 371) = 14.95, *p* < .001 | LM_Attribute (β = .12, *p* = .024)<br>LM_Person (β = .20, *p* < .001)<br>LM_Debilitating (β = –.13, *p* = .004) |
| Self-esteem | | | | | |
| | 1 | .54 | — | F(6, 375) = 74.10, *p* < .001 | Emotional Stability (β = .28, *p* < .001)<br>Extraversion (β = .19, *p* < .001)<br>Conscientiousness (β = .19, *p* < .001) |
| | 2 | .69 | .15 | F(4, 371) = 45.18, *p* < .001 | LM_Attribute (β = .11, *p* = .010)<br>LM_Person (β = .34, *p* < .001)<br>LM_Debilitating (β = –.15, *p* < .001) |

*Note.* For sample source, 0 = Undergraduate and 1 = CloudResearch; LM_Attribute = Lonely Attribute Mindset ; LM_Person = Lonely People Mindset ; LM_Enhancing = Loneliness as Enhancing Mindset; LM_Debilitating = Loneliness as Debilitating Mindset; LM_Attribute, LM_Person, and LM_Enhancing are coded such that higher scores indicate a stronger growth mindset; LM_Debilitating is coded such that a higher score indicates a stronger loneliness as debilitating mindset.
